# Exploring the Relationship between Cardiorespiratory Fitness and Executive Functioning in Adults with ADHD

**DOI:** 10.3390/brainsci13040673

**Published:** 2023-04-17

**Authors:** Michelle Ogrodnik, Sameena Karsan, Victoria Cirone, Jennifer J. Heisz

**Affiliations:** 1 Department of Kinesiology, Faculty of Science, McMaster University, Hamilton, ON L8S 4L8, Canada; 2 Department of Kinesiology and Health Sciences, Faculty of Health, University of Waterloo, Waterloo, ON N2L 3G1, Canada; 3 Department of Physical Therapy, Djavad Mowafaghian Centre for Brain Health, Vancouver Coastal Health Research Institute, The Centre for Aging SMART, The University of British Columbia, Vancouver, BC V1Y 1T3, Canada

**Keywords:** ADHD, adults, executive function, cardiorespiratory fitness

## Abstract

Objective: Associations between measures of executive functioning (EF) and cardiorespiratory fitness (CRF) were examined for adults with and without ADHD. Method: Measures of executive functioning including the Stroop task, Wisconsin Card Sorting task, and Operation Span Task were completed virtually (n = 36 ADHD; n = 36 Control). Participants completed the Six-Minute Walk Test to estimate CRF. Results: Mean performance measures of executive function did not differ by group. However, higher estimated CRF was associated with better Stroop task performance, and the association was strongest for individuals with ADHD. Conclusion: In adults with ADHD, higher estimated CRF was associated with better inhibitory control, but not with other measures of executive functioning.

## 1. Introduction

Attention deficit hyperactivity disorder (ADHD) is one of the most common neurodevelopmental disorders affecting approximately six percent of children and three percent of adults worldwide [1]. A core symptom of ADHD is executive dysfunction [2], which disrupts decision-making [3], sustained focus [4], and the ability to manage multiple competing demands [5]. Unfortunately, societal norms are built on the premise that everyone’s executive functioning is always operating optimally, and consequently, those with ADHD often experience real-world problems. For example, children with ADHD participate less in class, have a lower retention capacity for learning, and struggle to perform well on tests compared to their neurotypical peers [6]. These challenges in childhood can persist into adulthood, limiting future academic achievement and occupational attainment [7,8,9], and can negatively impact their quality of life [10,11]. Alongside societal shifts towards inclusivity, it is critical to consider immediate and accessible supports that can help people with ADHD manage their executive dysfunction. Most research on ADHD has focused on children and adolescents, even though the recent criteria for ADHD describe it as a chronic condition that often persists into adulthood [12,13]. Therefore, it is important to understand how to best support executive dysfunction in adults with ADHD, which was the aim of the present study.

Executive dysfunction associated with ADHD can arise from an impairment in one of three executive functions: inhibitory control, cognitive flexibility, or working memory. Inhibitory control refers to one’s ability to override an automatic or prepotent response to a stimulus [14], and has been described as the hallmark of executive dysfunction for those with ADHD [15]. Cognitive flexibility refers to one’s ability to transition accurately and efficiently between cognitively demanding tasks [16]. Working memory refers to one’s capacity to keep relevant information in mind when completing a cognitive task [17]. When compared to neurotypical children, those with ADHD perform worse on a range of tasks that depend on executive functions including having greater variability in reaction times on tasks of alertness, making more errors on tasks of distractibility, and having poorer overall cognitive flexibility [18]. Research on adults with ADHD parallel that of children, with some evidence indicating that executive dysfunction is a part of the chronic symptomology [19,20,21]. For example, adults with ADHD self-reported difficulty with cognitive flexibility and performed poorly on the Iowa Gambling Task [5], during which they struggled to select the optimal solution to a problem when multiple competing options were available and made more impulsive decisions (i.e., had faster reaction times) on questions that assessed risk-taking behaviour.

The most common intervention for ADHD symptoms is pharmacotherapy [22]. Approximately 60% of children diagnosed with ADHD take medication at some point to manage their symptoms [23]. The most prescribed ADHD medications are stimulants that increase dopamine levels in the brain, which the prefrontal cortex needs to optimally perform its suite of executive functions [12,24]. Pharmacotherapy is also the front-line treatment for adults with ADHD and it can improve many domains of executive function [25]; however, there are also multiple points of concern. Across the board, children and adults with ADHD have poor adherence to their prescribed medication. Over 60% of children failed to adhere to their prescribed medication schedule over five years [26] and approximately 18% of adults failed to adhere to their prescribed medication schedule over two weeks [27]. Unfortunately, when individuals do not take their medication, symptoms of executive dysfunction can return [28]. ADHD medication can also give rise to negative side effects including appetite suppression, insomnia, irritability, and increased blood pressure [29,30], and this is the main reason why most people discontinue medication use [31]. Finally, there are concerns regarding the long-term use of ADHD medication, including safety and efficacy [32]. Taken together, it is not surprising that only approximately seven percent of adults with ADHD take medication [33]. As such, there is a critical need for alternative strategies to help adults with ADHD manage their symptoms.

Physical activity has been identified as one of the best non-pharmacological interventions for managing ADHD symptoms [34,35]. Physical activity can increase levels of dopamine and improve prefrontal functioning [36,37], thus having a similar intended pharmacotherapeutic effect as ADHD medications [38]. An acute bout (~30 min) of physical activity can improve executive functioning in children [39,40,41] and adults with ADHD [42,43,44] as well as in neurotypical children and adults [45,46]; however, most of the research on physical activity for ADHD has been conducted in children.

The beneficial effects of an acute bout of physical activity for executive dysfunction in children are noteworthy. An acute bout of physical activity in children with ADHD has been documented to elicit significant improvements in inhibitory control, cognitive flexibility, and working memory [40,47,48,49], with some research reporting positive effects on inhibitory control after just five minutes of physical activity [50]. Although more research is needed in adults with ADHD [39], there is some evidence demonstrating that acute physical activity benefits executive functions [42,43]. College-aged students with ADHD were tested and found that a 30-min acute bout of jogging improved all three domains of executive function but elicited the greatest benefit for inhibitory control [42]. More recently, adults with ADHD completed a high-intensity interval cycling protocol for a total of 16 min. Following their protocol, adults with ADHD showed improvements in processing speed and decreased reaction time variability as measured by the AX-Continuous Performance Test, which assesses goal maintenance abilities and working memory [43].

With respect to the chronic effects of regular engagement in physical activity on executive functions, even less is known about the effects in adults with ADHD. Engaging in chronic physical activity can improve cardiorespiratory fitness (CRF), defined as the circulatory and respiratory systems’ ability to supply oxygen to the skeletal muscles for energy production [51]. Previous research has noted that neurotypical children and adults with higher CRF have better inhibitory control, cognitive flexibility, and working memory [52,53,54]. The same is true for children with ADHD [49,55,56,57]. In children with ADHD, 8 weeks of aerobic training resulted in better inhibitory control [58] and 12 weeks of aerobic training resulted in better inhibitory control, cognitive flexibility, and working memory [59]. In children and adolescents with ADHD, a 2023 meta-analysis highlighted that chronic exercise interventions have small-to-moderate effects on inhibitory control, working memory, and cognitive flexibility, with the greatest improvements for inhibitory control [57]. While these papers did not measure changes in fitness directly, improved CRF is a result of chronic physical activity, and prior research in neurotypical individuals points to CRF as a component of the mechanism through which physical activity improves executive functions [60,61].

In adults with ADHD, we are only aware of two prior studies that have examined the relationship between CRF and executive functions specifically. In one study, Jeoung (2014) explored the association between CRF and measures of self-reported executive dysfunction including inattention/memory, hyperactivity/restlessness, and impulsivity/emotional lability in men with ADHD. They found that those with higher CRF had fewer self-reported symptoms of executive dysfunction. However, this research is limited because it did not include women with ADHD, nor did it include objective assessments of executive functioning [62]. In the other study, Mehren and colleagues (2019) explored the association between CRF and inhibitory control performance on the Flanker Task in men and women with ADHD and found that those with higher CRF had better inhibitory control performance; however, this paper did not assess other two domains of executive function, namely, cognitive flexibility or working memory [44].

The present study was designed to fill these gaps by investigating the relationship between CRF and three domains of executive functioning in adults with ADHD. We examined cross-sectional differences between adults with and without ADHD on performance measures of inhibitory control, cognitive flexibility, and working memory. We used an objective estimate of CRF and assessed whether it predicted performance on each measure of executive function. We also explored whether the association between CRF and executive functions differed by group (ADHD vs. Control). It was hypothesized that adults with ADHD would have poorer performance across all three domains of executive functioning. It was also expected that participants with higher CRF would have better executive functioning across all three domains, but the associations would be stronger for adults with ADHD. Previous research reveals stronger relationships between fitness and cognition in individuals with greater executive dysfunction (i.e., older adults) compared to those with less executive dysfunction (i.e., neurotypical younger adults) [63]. Therefore, we hypothesized that adults with ADHD may have a stronger relationship between fitness and cognition because of their executive dysfunction. Since executive dysfunction has significant negative impacts on daily living [8] and adults with ADHD are understudied [39], the present work provides important baseline data for understanding an accessible way to potentially help manage ADHD symptoms.

## 2. Methods

### 2.1. Participants

Previous research using this data set, including an overview of the participants, is provided in Ogrodnik et al. (2023) [64]. This project was approved by the McMaster Research Ethics Board (Project ID 5111). Adults between 18 and 35 years old were recruited to complete this study (May 2020–February 2022) and they received monetary compensation. Participants needed to be able to read, write, and speak English. As the experimental protocol included the Stroop task (described below), participants were deemed ineligible if they had a known diagnosis affecting colour vision. Participants also could not have any other neurodevelopmental diagnoses beyond ADHD. Prior to the completion of the protocol, all participants were screened using the Physical Activity Readiness questionnaire to ensure safety for the six-minute walk test [65]. A self-reported formal diagnosis of ADHD was required for those in the ADHD group. However, all participants also completed the Connors Adult ADHD Rating Scale (CAARS) to ensure they met the appropriate cut-offs for their respective groups; participants in the ADHD group needed to score ≥ 65 while those in the neurotypical group needed to score < 65. Those taking ADHD-related medication needed to consent to refrain from taking their medication for 24 h prior to their virtual visit, to participate. As part of a broader study, a pre-established sample size of 71 participants was calculated (fixed model, R^2^ deviation from zero, Cohen’s f = 0.23, β = 0.95 and α = 0.05) using G*Power version 3.1.9.2 [66]. Eighty-five participants completed the study; however, due to missing data (n = 1; the participant was unable to complete the cognitive task portion of the experiment due to technical difficulties), no formal ADHD diagnosis (n = 1), or not meeting CAARS score cut-offs (control = 7; ADHD = 4), 72 participants were included in data analyses for this project (36 ADHD, 36 non-ADHD) [64].

### 2.2. Materials

#### 2.2.1. Conner’s Adult ADHD Rating Scale (CAARS)

The CAARS is a reliable and validated 30-item self-report questionnaire, which was used to evaluate ADHD symptoms in all participants [67,68]. Items were ranked using a 0–3 scale of “not at all, never” = 0, “just a little”, “once in a while” = 1, “pretty much, often” = 2, and “very much, very frequently” = 3. Responses were combined using guidelines from Multi-Health Systems, where (1) a clinical cut-off of 65 indicated the presence of ADHD and (2) a higher score reflected greater symptom severity.

#### 2.2.2. Estimated Cardiorespiratory Fitness (CRF)

The six-minute walk test (6MWT) is a tool used to measure the distance an individual can walk on a straight path in six-minutes [69,70]. The 6MWT was administered virtually using the ‘6WT’ mobile application [71] where participants walked outdoors, on their own, and the application automatically recorded their distance. Prior to completing the 6MWT, participants also measured their heart rate using the ‘Instant Heart Rate’ mobile application by Azumio, which has been validated against gold standard heart rate measures [72]. Taken together, the distance from the 6MWT, resting heart rate, and a participant’s sex, age, and body weight were used to calculate their VO_2_ max, which reflects an estimated maximum rate of oxygen consumption [73]. Estimated VO_2_ max was calculated through Equation (1) as this has been shown to be a valid and reliable measure “and accounts for approximately 72% of the variance associated with the gold-standard measure for cardiorespiratory fitness” [64,74,75].

Estimated VO_2_ max (mL · kg^−1^ · min^−1^) = 70.161 + (0.023 × 6MWT [m]) − (0.276 × weight [kg]) − (6.79 × sex, where m = 0, f = 1) − (0.193 × resting HR [beats per minute]) − (0.191 × age [y]) (1)

### 2.3. Cognitive Tasks

All cognitive tasks were administered virtually using Inquisit Web (Version 6.3.5). Participants were provided the web-license link by a research assistant and completed all tasks on their personal computer. All tasks used were from the Millisecond library [76].

### 2.4. Stroop

The Stroop task, version “Colour Word Stroop with Keyboard Responding” [77], was used to assess inhibitory control. Stimuli presented on the screen were either words identifying one of four colours (“red”, “green”, “blue”, “black”) or solid, coloured rectangles. The solid rectangles were used in control trials where participants were instructed to identify the colour of the rectangle. Colour words were used for congruent and incongruent trials. Congruent trials were when the ink colour matched the word (i.e., the word “red” presented in the colour red) while incongruent trials had mismatched ink colour (i.e., the word “red” presented in the colour black). Trials were randomly sampled, and participants were instructed to identify the colour of all trials as accurately and quickly as possible using the assigned keyboard responses. Stimuli remained on the screen until the participant responded. Following a response, a 200-millisecond interval was applied between all trials. In total participants completed 84 trials. Overall accuracy and accuracy on congruent and incongruent trials were recorded. Interference scores were calculated by taking the difference between incongruent and congruent performance for both accuracy and reaction time (RT).

### 2.5. Wisconsin Card Sorting Task (WCST)

The Wisconsin Card Sorting Task [78] was used to measure cognitive flexibility. Participants were instructed to sort a deck of cards into separate categories without being provided explicit instructions on the rules of categorization. Potential categories were based on shape, colour, or number of items on the face of the card. The cards remained on the screen until they were categorized. Participants reached the end of the task by completing the maximum number of trials, which included 2 decks of 64 cards, for a total of 128 cards, or by successfully completing 6 blocks of 10 correct trials for colour, shape, or number. The percent of correct trials and percent perseverative errors—i.e., using the previous categorization strategy despite a change in the rule—were calculated.

### 2.6. Operation Span (OSPAN) Task

The Operation Span Task [79] was used to measure working memory. For each trial, participants were presented with a sequence of three to seven letters on a computer screen for one second each and were instructed to remember them in order. Then, for up to five seconds, participants were required to review simple math equations with a provided answer and identify whether the provided answer was “true” or “false”. Finally, participants were presented with a three by four matrix of letters and had to select the letters in sequence as they had appeared prior to the math task. The matrix remained on the screen until the participant submitted a recall sequence. Participants completed four practice trials where the letter sequencing and math problem were carried out separately, and three practice trials where the letter sequencing and math problem were carried out together, as described above. Participants then began the recorded OSPAN test with a total of 15 trials. Performance on the OSPAN task was calculated for participants who scored higher than 85% on the math trials, by summing the total number of letter sequences that were perfectly recalled, where a higher score indicates superior working memory capacity.

### 2.7. Covariates

Factors of sex, sleep, and mental health were characterized and included as covariates because they are known to impact both CRF [80,81,82] and cognition [83,84,85]. Ad hoc questions were used to determine sex and sleep. Specifically, participants were asked “What sex were you assigned at birth?” with options of male, female, intersex or “prefer not to say”, and “How many hours of sleep did you get last night?”.

Mental health was characterized using the Depression, Anxiety, and Stress Scale 42 [86]. The DASS is a validated questionnaire that measures constructs including depression, anxiety, and stress, all graded on a 0–3 scale (“did not apply to me at all” = 0, “applied to me to some degree” = 1, “applied to me to a considerable degree” = 2, and “applied to me very much” = 3) with a higher score indicating more severe symptoms. As recommended in the DASS manual, an overall index of mental health for each participant was computed by averaging the average z-scores for each subscale [86].

### 2.8. Procedure

This study was completed via a virtual visit on Zoom (Version 5.11.10) that lasted approximately 2.5 h. Participants completed the DASS and CAARS questionnaires along with a demographic questionnaire that included questions about biological sex and sleep the night before. Then, participants completed the cognitive tasks via Inquisit Web, in the following order: (1) Stroop, (2) WCST, and (3) OSPAN. The researcher provided instructions prior to each task and remained on the Zoom call while participants completed each task independently. Finally, participants completed the estimated CRF protocol. As the first step, each participant rested for five minutes and took three measurements of their resting heart rate monitored by the researcher. Then, the researcher provided instructions on how to complete the 6MWT, including how to use the 6WT mobile application. Participants temporarily left the Zoom call to complete the 6WT outside, unsupervised. After finding an appropriate location to complete the test (i.e., flat path, limited tall buildings that may disrupt GPS signal), participants started the application, which automatically timed the six minutes and recorded the distance walked. The session ended after participants returned to the Zoom call to report their 6MWT distance and debriefed with the researcher.

### 2.9. Statistical Analysis

IBM SPSS (V28) was used for all data analyses, with an alpha level set to less than 0.05. Descriptive statistics were calculated, and normality was assessed for all measures outlined above. Normality was assessed using visual inspection alongside values of skewness and kurtosis, and the Shapiro–Wilk test. Outliers were considered if a data point exceeded three standard deviations beyond the mean [87]. Only one participant was removed from the ADHD group for all Stroop analyses because their average reaction time on incongruent trials was more than five standard deviations away from the mean.

Three domains of executive functioning (inhibitory control, cognitive flexibility, and working memory) were evaluated. Inhibitory control was evaluated using the Stroop task performance scores of overall accuracy, accuracy on incongruent trials, and interference scores. Cognitive flexibility was evaluated using the WCST percent perseverative errors and percent of correct trials. Working memory was evaluated using the OSPAN score.

Continuous variables (domains of executive functioning and estimated CRF) were compared between groups using independent samples *t*-tests. Exploratory moderation analyses were carried out using Model 1 in Hayes PROCESS Macro, Version 4.1 SPSS plug-in, to test the associations between CRF (IV) and measures of executive function (DVs), and to explore whether the associations were moderated by group. Covariates in all models included sex, sleep, and mental health.

## 3. Results

### 3.1. Descriptive Statistics

Most participants were white (49%) and female (75%) and had an average age of 21 years old (SD = 3.0) (see Table 1).

As expected, CAARS scores were significantly higher for the ADHD group (M = 79.86, SD = 7.00) than the controls (M = 49.14, SD = 8.29; *p* < 0.001). Mental health, as measured by the DASS, was significantly worse for the ADHD group (Mean Z score = 0.41, SD = 0.98) than for controls (Mean Z score = −0.41, SD = 0.60; *p* < 0.001) (see Ogrodnik et al., 2023, for more details on mental health outcomes) [64]. The groups did not differ in estimated CRF (*p* = 0.43) or in their performance on any task of executive functioning (all *p* ≥ 0.17) (Table 2).

### 3.2. Fitness x Executive Function

**Figure 1:**
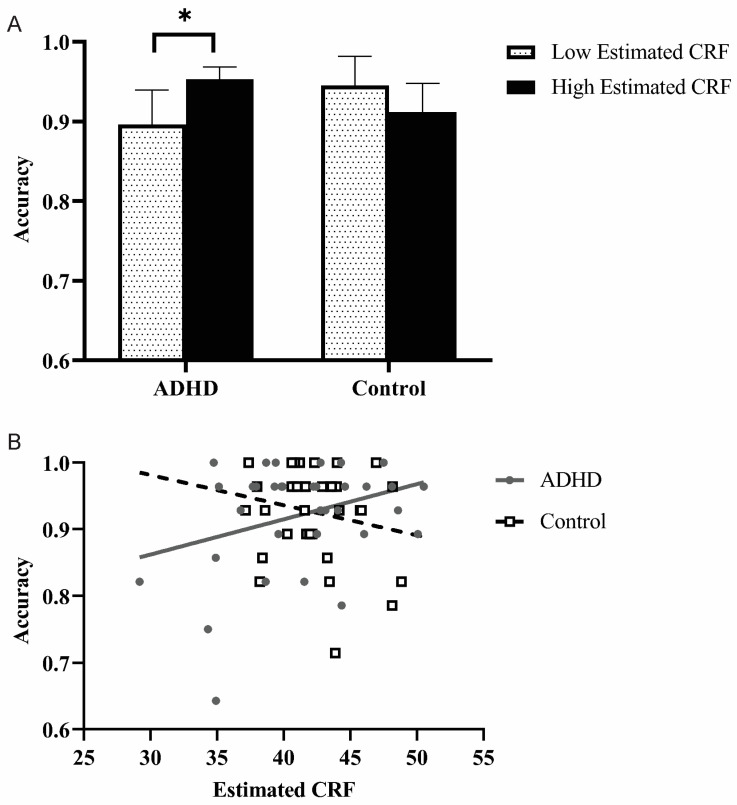
(**A**) The moderating effect of group (ADHD vs. Control) on the relationship between estimated CRF and Stroop incongruent accuracy. Bars reflect standard error. * *p* < 0.05 (**B**) Scatterplot of raw data included in the mediation analysis. Regression lines reflect the mediation output (solid grey line = ADHD; dashed black line = Control).

Higher estimated CRF was associated with greater accuracy on *all* trials of the Stroop task (*p* < 0.05), and this relationship was observed for those with and without ADHD (*b* = 0.01, *SE b* = 0.003, 95% CI = 0.00 to 0.01). Furthermore, there was an association between higher estimated CRF and greater accuracy on *incongruent* trials of the Stroop task, but this was moderated by group (Table 3), and only observed for adults with ADHD (*b* = −0.01, *SE b* = 0.01, 95% CI = −0.02 to −0.001) (Figure 1A,B). No other associations were observed between estimated CRF and executive functioning (Table 3).

## 4. Discussion

The current project examined the association between estimated CRF and executive functioning in adults with ADHD compared to neurotypical controls. To our knowledge, this was the first study to examine this association in an adult group across all three domains of executive functioning. Surprisingly, both groups were well-matched in terms of their estimated CRF and executive functioning. Importantly, higher estimated CRF was associated with better inhibitory control for adults with ADHD.

A strength of our study is that we used three validated computerized tests of executive functions that are commonly used to assess deficits in inhibitory control [88], cognitive flexibility [89], and working memory [90,91]; namely, the Stroop task measured inhibition, the Wisconsin Card Sorting task measured cognitive flexibility, and the OSPAN task measured working memory. Yet, in our adult sample, we did not observe group differences between adults with and without ADHD in performance on any of these objective measures. This contrasts some prior research showing that children and adults with ADHD perform worse on these tasks compared to their neurotypical peers with small-to-moderate effects [18,19,20,21]. That said, the effect sizes tend to be larger in children than in adults [92] The more subtle impairments in executive task performance for adults with ADHD may explain why not all research, including ours, observe significant group differences. For example, in line with our observations, Barkley and Murphy (2011) [93] found no significant group differences in the proportion of adults with and without ADHD who showed clinical impairments on the Stroop task of inhibition, the Wisconsin Card Sorting Task of cognitive flexibility, and the Digit Span task of working memory [93].

Individual differences among adults with ADHD may further minimize group-wise differences. A key difference between our study and prior studies is that our participants were highly educated. Most of our participants were in pursuit of or held a university degree and may have already developed effective strategies for managing their executive dysfunction [13,94,95]. Indeed, previous research that used the same cognitive tasks found that adults with ADHD and a higher IQ had less pronounced differences in inhibitory control and working memory performance—these differences were still evident in adults with ADHD and a standard IQ [96]. Furthermore, in adults with ADHD, those with a standard IQ tend to have lower academic grades, educational attainment, and occupational attainment than those with a higher IQ [97]. Therefore, the lack of a group-wide ADHD deficit in the executive function tasks observed here may be a consequence of our highly educated, adult sample.

That said, individual differences in inhibitory control performance were observed among adults with ADHD when CRF was accounted for. This observation further supports the importance of acknowledging the heterogeneity in executive dysfunction among adults with ADHD. In the present study, we found that CRF was associated with accuracy on incongruent trials in adults with ADHD. Importantly, this was not observed for neurotypical participants, though higher CRF was associated with better overall accuracy on the Stroop task for all participants. Our results align with previous research which suggests that fitness-related benefits may be most useful for those with executive dysfunction, whereas healthy controls may not show notable benefits given the ceiling effects [44,55,98]. Furthermore, the association between higher CRF and better performance for those with ADHD was not observed for the other tasks of executive functions. This aligns with previous research in children and adolescents with ADHD, which has found that fitness has the strongest impact on inhibitory control compared to other executive functions, including working memory [55,99]. Our research suggests the same is true in adults with ADHD. While it is unclear why inhibitory control may have a stronger connection with CRF in comparison to other executive functions, there seems to be a selective association between inhibitory control and other health outcomes. For example, prior studies have documented a selective link between tasks of inhibitory control and adiposity that was not observed for other cognitive tasks [100,101]. From a physiological perspective, this could be because cardiometabolic health is associated with enhanced cerebral vasculature and neurotrophic pathways along with increased cerebral blood flow [36,37,102,103]. Thus, the brain would be more adequately supplied with the resources it needs to perform the metabolically demanding tasks of inhibitory control. This may be particularly important for people with ADHD who display hypoperfusion of the prefrontal cortex [104]. From a behavioural perspective, this could be because inhibitory control is “flexed” every time one engages in a healthy behaviour that requires self-control. In fact, previous research has noted that inhibitory control training can positively impact health behaviours [105]. Although our cross-sectional study is unable to test these hypotheses, they should be evaluated by future research.

While not seen in our data, adults with ADHD tend to be less physically active than their neurotypical peers [106,107], and therefore, may especially benefit from an exercise intervention that increases fitness. Given that inhibitory control is the hallmark of executive dysfunction associated with ADHD [108] and may be especially sensitive to chronic exercise adaptations [109], exercise programs that increase CRF in adults with ADHD may be supportive of their inhibitory control functioning and should be explored in future research. This may also benefit beyond inhibitory control; associations between ADHD and physical conditions have been documented including type II diabetes, metabolic syndrome, and obesity [110,111], many of which have symptoms that can be managed with exercise [112].

In contrast to the objective measures of executive dysfunction, we observed the expected significant group differences in *subjective* ratings of ADHD symptoms, as indicated by higher CAARS scores for the ADHD group than controls. This demonstrates a divergence between subjective and objective assessments of cognition, which further complicates the assessment of executive dysfunction in adults with ADHD. Although some studies in children with ADHD have found that subjective and objective measures of executive function are moderately correlated [113,114,115], there are also documented inconsistencies between subjective and objective measures for children with ADHD [114,116]. Our work extends these inconsistencies into an adult population and highlights the need for multiple forms of assessments to fully capture executive dysfunction in adults with ADHD [117,118].

The lack of concordance between subjective and objective measures of executive dysfunction may reflect differences in the nature of the dysfunction being captured by the assessments. Self-reported assessments ask participants to reflect on their daily life, which tends to be more ambiguous and complex than performance-based measures using highly controlled tasks with a unitary predefined goal. Like our results, a prior study found that adults with ADHD self-report worse executive dysfunction than their performance on objective measures would indicate [119]. Similar findings are observed in children with autism spectrum disorder, another neurodevelopmental disorder that causes difficulty with executive functioning; specifically, the findings suggest that executive functioning deficits in autism are worse when assessing their “day-to-day executive function-related behaviour” than their in-lab test performance [120]. In adults with ADHD, Barkley and Murphy (2010; 2011) [93,121] suggest that perceptions of executive dysfunction, rather than tasks of executive function, are more closely related to real-life impairments, including academic and occupational outcomes [93,121]. As such, future research should consider a particular emphasis on using assessments that simulate daily tasks (i.e., poor self-organization and sustained effort in everyday settings) to capture the more subtle struggles of executive dysfunction that adults with ADHD grapple with daily [122].

### Limitations and Future Directions

The current data provide insight on executive dysfunction in adult ADHD and its association with CRF. While this study fills an important gap in the literature, it is not without limitations. Most importantly, our data are cross-sectional, and therefore, causality cannot be determined. While previous research in children with ADHD has established that higher fitness is associated with better executive functioning [55] and that chronic exercise interventions can improve executive functioning [58,123], future randomized controlled trials are needed to confirm the direction of this relationship in adults with ADHD. It is also important for future research to consider other factors that may exacerbate executive dysfunction in ADHD including chronic stressors associated with lower socioeconomic status [124,125,126,127].

All data were collected during the COVID-19 pandemic and, therefore, it is important to acknowledge the potential for measurement errors within our virtual protocol. For example, CRF was estimated using the Six-Minute Walk Test. Although this standardized test shows good validity [74], it was carried out unsupervised. Likewise, participants completed all cognitive tasks in their own environments. The lack of consistency (e.g., environmental distractors, different device sizes, internet lags, etc.) may have added noise to these measurements. While the virtual nature may have introduced additional variance, it is interesting that participants across both groups performed well on all tasks. Given that there were no group-level differences in executive functioning, the executive dysfunction associated with ADHD may vary across the lifespan and depend on factors such as education level. Therefore, more work specifically in adults is needed.

## 5. Conclusions

Taken together, our results provide important insights into the executive dysfunction of adults with ADHD and its association with CRF. Although at the group level adults with ADHD performed similarly to the controls on the tasks of executive function, we observed interesting individual differences, such that higher fitness in adults with ADHD was associated with better inhibitory control. The same individual differences were not observed for cognitive flexibility or working memory. Given that inhibitory control is the hallmark of executive dysfunction for ADHD [15], using physical activity to improve fitness in adults with ADHD may be an effective strategy to support symptom management. However, it should be acknowledged that adhering to a program of physical activity requires executive functioning to schedule and initiate [128], and therefore, future research is needed to identify the unique ADHD-related barriers that individuals may face so that the necessary support can be provided.

## Figures and Tables

**Table 1:** Demographic Characteristics.

Characteristic	ADHD		Control	
	N (%)		N (%)	
*Sex*				
Female	26 (72)		28 (78)	
Male	10 (28)		8 (22)	
Intersex	-		-	
*Gender*				
Woman	23 (64)		27 (75)	
Man	11 (30)		8 (22)	
Non-binary	2 (6)		1 (3)	
Two-spirit	-		-	
*Race*				
Caucasian	19 (53)		16 (44)	
East/Southeast Asian	5 (14)		8 (22)	
South Asian	4 (11)		7 (19)	
Multiracial	4 (11)		3 (10)	
Middle Eastern	2 (5)		2 (5)	
Black	1 (3)		-	
Latino	1 (3)		-	
Indigenous	-		-	
*Highest level of education*				
Less than secondary	-		1 (3)	
Secondary	21 (58)		26 (72)	
More than secondary	15 (42)		9 (25)	

**Table 2:** Cognitive Outcomes and CRF Outcomes.

Outcomes	ADHD Mean (±SD)	Control Mean (±SD)	*p*-Value
CAARS Scores	79.86 (7.00)	49.14 (8.29)	<0.001 ***
DASS Z Score	0.41 (0.98)	−0.41 (0.60)	<0.001 ***
Estimated CRF	41.55 (5.23)	42.35 (2.97)	0.43
*Stroop Task*			
Overall Accuracy	0.95 (0.04)	0.96 (.03)	0.47
Incongruent Accuracy	0.92 (0.08)	0.93 (0.07)	0.82
Accuracy Interference	−0.05 (0.08)	−0.06 (0.07)	0.68
Reaction Time Interference	223.2 (155.17)	284.63 (209.20)	0.17
*WCST*			
Percent Perseverative Errors	31.82 (18.34)	35.71 (21.63)	0.41
Percent Correct	71.96 (11.25)	71.26 (11.28)	0.79
*OSPAN*	37.81 (18.54)	39.89 (21.13)	0.66

Notes: CRF = Cardiorespiratory Fitness, WCST = Wisconsin Card Sorting Task, OSPAN = Operation Span task, *** *p* < 0.001. All analyses included N = 36 for the ADHD group and N = 36 for the Control group except Stroop task analyses which included N = 35 for ADHD and N = 36 for the Control group.

**Table 3:** Regression coefficients of the moderating effect of group (ADHD vs. Control) on estimated CRF and cognitive outcomes.

DV		R^2^	b	SE b	95% Cis	*p*
Stroop						
		0.16				0.07
Overall Accuracy	Estimated CRF		0.01	0.003	(0.00, 0.01)	<0.05 *
	Group		0.13	0.10	(−0.08, 0.33)	0.21
	Interaction		−0.003	0.002	(−0.01, 0.001)	0.22
		0.11				0.26
Incongruent Accuracy	Estimated CRF		0.02	0.01	(0.004, 0.03)	0.01 *
	Group		0.47	0.21	(0.05, 0.89)	0.03 *
	Interaction		−0.01	0.01	(−0.02, −0.001)	0.03 *
		0.07				0.57
Accuracy Interference	Estimated CRF		0.01	0.01	(−0.001, 0.03)	0.06
	Group		0.38	0.22	(−0.05, 0.82)	0.08
	Interaction		−0.01	0.01	(−0.02, 0.001)	0.07
		0.10				0.34
RT Interference	Estimated CRF		8.08	17.30	(−26.47, 42.64)	0.64
	Group		36.11	526.41	(−1015.52, 1087.74)	0.95
	Interaction		0.14	12.46	(−24.76, 25.04)	0.99
WCST						
		0.04				0.82
Percent Perseverative Error	Estimated CRF		0.02	1.83	(−3.64, 3.67)	0.99
	Group		11.25	57.21	(−103.01, 125.52)	0.84
	Interaction		−0.21	1.35	(−2.91, 2.49)	0.88
		0.08				0.46
Percent Correct	Estimated CRF		0.01	0.01	(−0.01, 0.03)	0.58
	Group		0.17	0.31	(−0.45, 0.80)	0.58
	Interaction		−0.005	0.01	(−0.02, 0.01)	0.52
OSPAN						
		0.03				0.94
OSPAN	Estimated CRF		−0.50	1.82	(−4.14, 3.14)	0.79
	Group		−5.73	57.03	(−119.63, 108.17)	0.92
	Interaction		0.23	1.35	(−2.46, 2.92)	0.87

Notes: CRF = Cardiorespiratory Fitness, WCST = Wisconsin Card Sorting Task, OSPAN = Operation Span task, * *p* < 0.05. All analyses included N = 36 for the ADHD group and N = 36 for the Control group except Stroop task analyses which included N = 35 for ADHD and N = 36 for the Control group.

## Data Availability

The data presented in this study are available on request from the corresponding author. The data are not publicly available as participants did not consent to this.
