# Correction: Ogrodnik et al. Exploring the Relationship between Cardiorespiratory Fitness and Executive Functioning in Adults with ADHD. *Brain Sci.* 2023, *13*, 673

**DOI:** 10.3390/brainsci13111518

**Published:** 2023-10-26

**Authors:** Michelle Ogrodnik, Sameena Karsan, Victoria Cirone, Jennifer J. Heisz

**Affiliations:** 1 Department of Kinesiology, Faculty of Science, McMaster University, Hamilton, ON L8S 4L8, Canada; 2 Department of Kinesiology and Health Sciences, Faculty of Health, University of Waterloo, Waterloo, ON N2L 3G1, Canada; 3 Department of Physical Therapy, Faculty of Medicine, University of British Columbia, Vancouver, BC V6T 1Z3, Canada; vcirone@student.ubc.ca; 4 Djavad Mowafaghian Centre for Brain Health, Vancouver Coastal Health Research Institute, Vancouver, BC V6T 1Z3, Canada; 5 Centre for Aging SMART at Vancouver Coastal Health, Vancouver Coastal Health Research Institute, Vancouver, BC V5Z 1M9, Canada

During the final proofreading step of this paper [1], some errors in the main text were introduced.

## Affilliation Correction

The original Affiliation 3 should be changed to Affiliations 3, 4 and 5. Victoria Cirone ^3^ in author name part should also be changed to Victoria Cirone ^3,4,5^.

Original Affiliation 3: Department of Physical Therapy, Djavad Mowafaghian Centre for Brain Health, Vancouver Coastal Health Research Institute, The Centre for Aging SMART, The University of British Columbia, Vancouver, BC V1Y 1T3, Canada.

After changed: 

Affiliation 3: Department of Physical Therapy, Faculty of Medicine, University of British Columbia, Vancouver, BC V6T 1Z3, Canada.

Affiliation 4: Djavad Mowafaghian Centre for Brain Health, Vancouver Coastal Health Research Institute, Vancouver, BC V6T 1Z3, Canada.

Affiliation 5: Centre for Aging SMART at Vancouver Coastal Health, Vancouver Coastal Health Research Institute, Vancouver, BC V5Z 1M9, Canada.

## Error in Table

In the original publication, there was a formatting error in Table 3 as published. In the first row of identifying variables, it should be updated from Cis to CIs (both the C and I should be capitalized). The correction appears below. The authors state that the scientific conclusions are unaffected.
**DV**
**R^2^****b****SE b****95% CIs*****p***

## Text Correction

There were minor typographical errors in the original publication. Below is the list of minor amendments. The authors state that the scientific conclusions are unaffected.

1. TYPO: Section 2. Methods; 2.1. Participants: please update “recruited to complete this study (May 2020–February 2022) and they...” from May 2020 to May 2021.

CORRECTION: Adults between 18 and 35 years old were recruited to complete this study (May 2021–February 2022) and they received monetary compensation.

2. TYPO: Section 2. Methods; 2.2. Materials; 2.2.2. Estimated Cardiorespiratory Fitness: please update “The six-minute walk test (6MWT) is a tool used to measure the distance an individual can walk on a straight path in six-minutes” by removing the dash from the six minutes at the end of the sentence. It should read: “The six-minute walk test (6MWT) is a tool used to measure the distance an individual can walk on a straight path in six minutes”.

CORRECTION: The six-minute walk test (6MWT) is a tool used to measure the distance an individual can walk on a straight path in six minutes [69,70].

3. TYPO: Section 2. Methods; 2.6. Operation Span (OSPAN) Task: please update “with a three by four matrix of letters” to “with a three-by-four matrix of letters”.

CORRECTION: Finally, participants were presented with a three-by-four matrix of letters and had to select the letters in sequence as they had appeared prior to the math task.

4. TYPO: Section 4. Discussion (paragraph 2): There is a missing period after reference 92.

CORRECTION: That said, the effect sizes tend to be larger in children than in adults [92].

The authors state that the scientific conclusions are unaffected. This correction was approved by the Academic Editor. The original publication has also been updated.
